# Identification of the DNA Replication Regulator MCM Complex Expression and Prognostic Significance in Hepatic Carcinoma

**DOI:** 10.1155/2020/3574261

**Published:** 2020-09-09

**Authors:** Ting Cao, Shi-jie Yi, Li-xin Wang, Juan-xia Zhao, Jiao Xiao, Ni Xie, Zhi Zeng, Qi Han, Hai-ou Tang, Yu-kun Li, Juan Zou, Qing Wu

**Affiliations:** ^1^Department of Digestive Medical, The Affiliated Nanhua Hospital, University of South China, Hengyang 421002, China; ^2^Department of Gastrointestinal Surgery, The Affiliated Nanhua Hospital, University of South China, Hengyang 421002, China; ^3^Center for Traditional Chinese Medicine and Immunology Research, School of Basic Medical Sciences, Shanghai University of Traditional Chinese Medicine, 1200 Cai Lun Rd., Shanghai 201203, China; ^4^Department of Pathology, The Affiliated Nanhua Hospital, University of South China, Hengyang 421002, China; ^5^Department of Endocrinology, The Affiliated Nanhua Hospital, University of South China, Hengyang 421002, China; ^6^Department of Pathology, Xianning Central Hospital, The First Affiliated Hospital of Hubei University of Science and Technology, Xianning 437000, China; ^7^Department of Oncology, Xianning Central Hospital, The First Affiliated Hospital of Hubei University of Science and Technology, Xianning 437000, China; ^8^Jishou University College of Medicine, Jishou 416000, China; ^9^Key Laboratory of Tumor Cellular and Molecular Pathology, College of Hunan Province, Cancer Research Institute, University of South China, Hengyang, Hunan 421001, China

## Abstract

**Background:**

The microliposome maintenance (MCM) complex, MCM2-7, is revealed to be involved in multiple cellular processes and plays a key role in the development and progression of human cancers. However, the MCM complex remains poorly elaborated in hepatic carcinoma (HCC).

**Methods:**

In the study, we found the mRNA and protein level by bioinformatics. We also explored the prognostic value, genetic alteration, interaction network, and functional enrichment of MCM2-7. The MCM expression and correlation among these MCMs in HCC cell lines were identified by western blot.

**Results:**

MCM2-7 was significantly increased in HCC tissues compared to normal liver tissues. The high level of MCM2-7 had a positive correlation with poor prognosis. However, MCM2-7 alterations were not correlated with poor OS. MCMs were both increased in HCC cell lines compared to the normal hepatocyte cell line. Furthermore, the positive correlation was found among MCMs in HCC cell lines.

**Conclusions:**

The MCM complex was increased in HCC tissues and cell lines and negatively correlated with prognosis, which might be important biomarkers for HCC.

## 1. Introduction

Hepatic carcinoma (HCC), a severe malignant disease of the digestive system, ranks sixth in terms of morbidity (over 0.8 million new cases) and fourth in mortality overall (over 0.7 million deaths) in 2018 [1]. There are two important risk factors for HCC, including alcohol consumption [2] and hepatitis virus [3]. Although the development of new targeted drugs and multidrug combinations has improved the battlefield for HCC, only about 5-14 percent of patients with HCC have a five-year survival rate [4, 5], with the development of bioinformatics and high-throughput sequencing technology, inhibitor of apoptosis protein (IAP) family members [6], kinesin family members [6], cytochrome P2C (CYP2C) subfamily members [7], CDK1, PBK, RRM2, and ASPM [8]. Recently, Han et al. found through bioinformatics that SCAMP3 may be an important marker in the development of liver cancer [9]. Therefore, it is very important to explore the correlation between prognostic value and new gene complexes in HCC.

The microliposome maintenance (MCM) protein was first discovered in sacs cerevisiae, and the mutants showed defects in microliposome maintenance, which play a key role in DNA replication [10]. MCM2-7 is a group of six structurally related proteins, from yeast to humans, which are highly conserved and interact to form a hexamer. In the progression of DNA synthesis, MCM2-7, as nuclear proteins, binds to chromatin by a cell cycle specific manner, resulting in promoting cell proliferation and helicase activity [10]. Disorders in these proteins can directly disrupt the DNA replication system, leading to cancer occurrence, development, and progression [11].

The MCM complex acts as an important regulator in multiple pathophysiological processes, including DNA replication [12], cell cycle [11], proliferation [13], migration [14], invasion [14], immune response [15], and apoptosis [11]. It has been previously reported in the literature that MCM is highly expressed in many cancers compared to normal tissues. For example, MCM2 got a high level in HCC, oral squamous cell carcinoma [16], gastric cancer [17], breast cancer [18], colon cancer [19], and ovarian cancer [20]. MCM2 was considered as a potential therapeutic target for cancer treatment, and the level of MCM2 could predict poor prognosis for osteosarcoma [21], gastric cancer [22], lung adenocarcinoma [23], diffuse large B cell lymphoma [24], and esophageal cancer [25]. Recent research suggested that MCM2 might be a potential therapeutic target for HCC [26]. Furthermore, Deng et al. found that MCM2 inhibition could increase the sensitivity of carboplatin in ovarian cancer cell [27]. MCM3 had similarly a high expression level in multiple cancer types, such as osteosarcoma [21], salivary gland tumors [28], and glioma [29]. Ha et al. indicated that MCM3 got a high expression in leukemia, lymphoma, uterine cervix cancer, colon cancer, lung cancer, gastric cancer, kidney cancer, breast cancer, and malignant melanoma [30]. MCM4 levels were elevated in esophageal cancer [31], uterine cervical carcinoma [32], and non-small-cell lung cancer [33]. MCM5, as another regulator in DNA replication, was overexpressed in colon cancer [19], oral squamous cell carcinoma [34], cervical cancer [35], thyroid cancer [36], and bladder cancer [37]. The expression of MCM6 was found to be enhanced and its high level had a close relationship with unfavorable prognosis in colorectal cancer [38], breast cancer [39], AO [15], HCC [14], endometrioid adenocarcinoma [40], lung cancer [41], meningiomas [42], cervical cancer [43], Hodgkin's lymphoma [44], and Merkel cell carcinoma [45]. Ectopic expression of MCM7 has been indicated that can promote the progression of prostate cancer [46], HCC [47], breast cancer [48], and acute myeloid leukemia [49].

These studies sufficiently indicated differential level of MCMs in multiple cancer types, but few studies systematically focused on the prognostic value of the whole MCM complex members in carcinogenesis. In this study, we comprehensively demonstrated the transcriptional level of MCMs and found its prognostic value in HCC. Moreover, we also analyzed the interaction network, genetic mutation, and functional enrichment of MCMs by bioinformatics.

## 2. Materials and Methods

### 2.1. Oncomine Analysis

Oncomine (https://www.oncomine.org) is a free access website to facilitate genome-wide expression analysis. We analyzed the transcriptional levels of MCM information in HCC from the Oncomine database [50].

### 2.2. GEPIA Analysis

Gene Expression Profiling Interactive Analysis (GEPIA) (http://gepia.cancer-pku.cn/), based on TCGA and GTEx data, is a web-based tool that delivers rapid customization to evaluate the relationship between MCM expression and staging in HCC [51].

### 2.3. Human Protein Atlas Analysis

The Human Protein Atlas (HPA) (https://www.proteinatlas.org/), an open online database of protein expression profiles, assists researchers in studying the differential expression of proteins between cancer and normal tissues.

### 2.4. Clinical Samples

A total of 30 HCC tissues were surgically resected in the Affiliated Nanhua Hospital, University of South China (Hengyang, Hunan, China), from 2010 to 2014. These tissues were made to 3 pieces of 10 × 10 chips. The collection and use of tissues followed the procedures according to the ethical standards as formulated in the Helsinki Declaration. And written informed consent was obtained from each patient, which was approved by the research ethics committee of University of South China. All patients did not receive radiotherapy or chemotherapy.

### 2.5. Kaplan-Meier Plotter Analysis

KM plotter (http://kmplot.com/analysis/), a survival database of patients, can analyze survival curves for many types of cancer [6]. The KM plotter is utilized to assess the prognostic value of MCMs in HCC.

### 2.6. GeneMANIA and STRING Analysis

GeneMANIA (http://genemania.org) [52] and STRING (https://string-db.org/) [53] are web tools to identify the interactions between genes/proteins, respectively. The interactions between MCMs and other gene/proteins are utilized by GeneMANIA and STRING at the gene or protein level.

### 2.7. cBioPortal for Cancer Genomics Analysis

cBioPortal for Cancer Genomics (http://www.cbioportal.org/), an open and free web tool, can be used to interactively explore multiple cancer genome datasets [54]. The correlation between MCM alterations and survival outcome in patients with HCC was analyzed by cBioPortal.

### 2.8. Metascape Analysis

Metascape (http://metascape.org) is an online analysis website available at enrichment pathway analysis and gene function annotation, which can be used to analyze the pathway and process enrichment of MCMs and the 40 neighboring genes [55].

### 2.9. Cell Culture

Five human HCC cell lines (HepG2, SNU-354, Huh 7, SNU-739, and HLF) and a normal human liver cell line (HL-7702) were cultured in Dulbecco's Modified Eagle Medium (DMEM; Gibco) containing 10% fetal bovine serum (FBS), 100 U/mL penicillin, and streptomycin, maintained at 37°C in a humidified atmosphere containing 5% CO_2_.

### 2.10. Quantitative Reverse Transcription Polymerase Chain Reaction (PCR)

PCR was conducted as previously described. Primers used were listed as follows: GAPDH forward GTCTCCTCTGACTTCAACAGCG, GAPDH reverse ACCACCCTGTTGCTGTAGCCAA; MCM2 forward TGCCAGCATTGCTCCTTCCATC, MCM2 reverse AAACTGCGACTTCGCTGTGCCA; MCM3 forward CGAGACCTAGAAAATGGCAGCC, MCM3 reverse GCAGTGCAAAGCACATACCGCA; MCM4 forward CTTGCTTCAGCCTTGGCTCCAA, MCM4 reverse GTCGCCACACAGCAAGATGTTG; MCM5 forward GACTTACTCGCCGAGGAGACAT, MCM5 reverse TGCTGCCTTTCCCAGACGTGTA; MCM6 forward GACAACAGGAGAAGGGACCTCT, MCM6 reverse GGACGCTTTACCACTGGTGTAG; and MCM7 forward GCCAAGTCTCAGCTCCTGTCAT, MCM7 reverse CCTCTAAGGTCAGTTCTCCACTC.

### 2.11. Immunohistochemistry

According to the manufacturer's instructions (Maixin Biotech. Co., Fuzhou, China), the slides were incubated with the primary antibody (diluted 1 : 100) at 4°C overnight, and normal rabbit immunoglobulin G was the negative control. The score of positive staining degree and percentage of stained cells were as follows: 0, no staining; 1, light brown; 2, dark brown and 0, stained cells < 5%; 1, stained cells range from 5% to 25%; 2, stained cells ranged from 26% to 50%; 3, stained cells > 50%. Scores were obtained by increasing the strength and reactivity of the reaction. A score of 2 is defined as high expression, and a score below 2 is defined as low expression.

### 2.12. Western Blot

The primary antibodies used in this study against MCM2 (ab31159), MCM3 (ab128923), MCM4 (ab4459), MCM5 (ab75975), MCM6 (ab201683), MCM7 (ab52489), and GAPDH (ab181603) were obtained from Abcam (Cambridge, MA, USA). Western blotting was conducted according to our previous report [56].

### 2.13. Statistical Analysis

Statistical analyses were performed in the R Programming Language (version 3.6). All statistical tests were bilateral, and *P* < 0.05 was statistically significant.

## 3. Results

### 3.1. The mRNA and Protein Expression of MCMs in HCC

**Figure 1:**
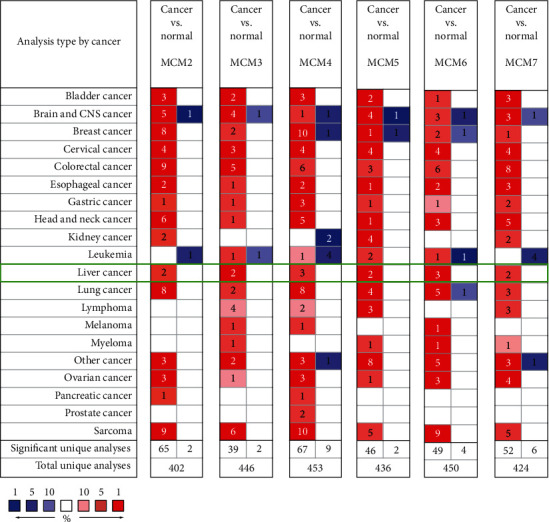
Oncomine analysis of MCMs in different cancer types. MCM expression between different cancer types and corresponding normal tissues was summarized. Threshold (*P* value ≤ 0.05; ∣FDR | ≥2; gene rank ≤ 10%; data type: mRNA) is expressed in colored cells. In tumor tissue, red cells represent overexpression of the target gene compared to normal tissue, while blue cells represent downregulation of the gene. Gene levels are indicated by the color depth of the cell. (You can refer to the web version of this article to explain the color reference in the diagram.)

Firstly, we utilized the Oncomine database to extract the data of MCM transcriptional levels in different cancer and corresponding normal tissues (Figure 1). The datasets of total unique analyses for MCM2, MCM3, MCM4, MCM5, MCM6, and MCM7 were 402, 446, 453, 436, 450, and 424, respectively. In cancer datasets, these MCMs were increased in most cancers, especially in bladder cancer, brain and CNS cancer, breast cancer, cervical cancer, colorectal cancer, esophageal cancer, gastric cancer, head and neck cancer, HCC, lung cancer, ovarian cancer, and sarcoma. Furthermore, the level of MCM2 in cancer tissues was increased in 65 datasets and decreased in 2 datasets compared to normal tissues. The MCM3 level was significantly enhanced in 39 datasets but reduced in 2 datasets. For MCM4, 67 datasets indicated overexpression, but 9 datasets indicated low expression. The mRNA level of MCM5 was upregulated in 46 datasets but downregulated in 2 datasets. High expression of MCM6 was observed in 49 datasets, while low expression was detected in 4 datasets. Moreover, the increased level of MCM7 was found in 52 datasets, but decreased level was observed in 6 datasets.

MCM4 was also enhanced in HCC compared to normal tissues based on Wurmbach Liver datasets [3]. Chen Liver datasets showed an obviously increased MCM6 level in HCC [57]. In addition, Roessler Liver and Roessler Liver 2 datasets indicated that MCMs were both significantly increased in HCC compared to normal tissues [58]. The statistical significance results with corresponding *P* values are shown in Figure 1 and Table 1.

**Figure 2:**
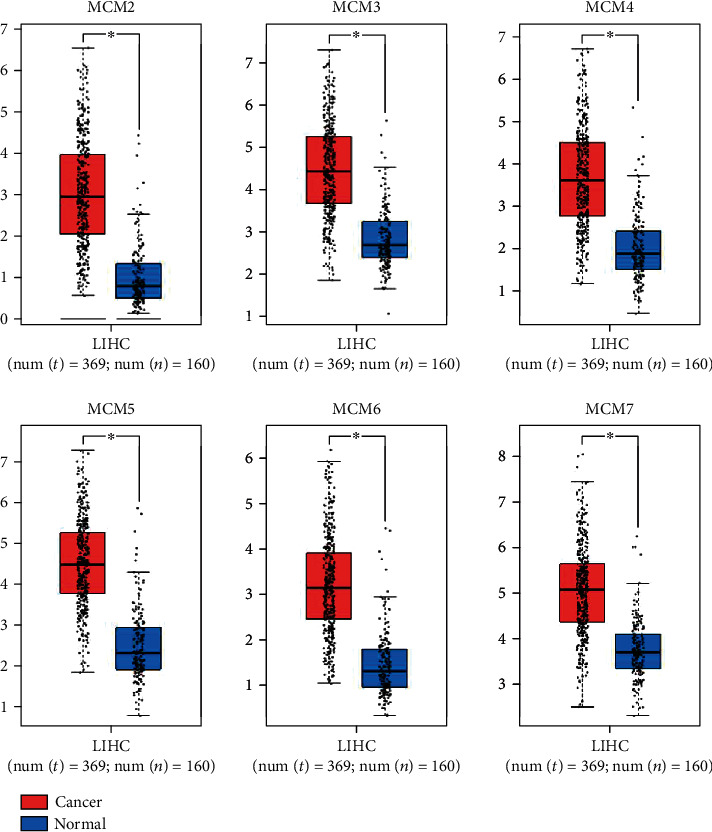
The mRNA expression levels of MCMs by GEPIA analysis in HCC. Box plots of individual MCM level in HCC tissues and normal liver tissues, *P* value ≤ 0.05.

We also used GEPIA to compare the transcriptional levels of MCMs in HCC and normal tissue (Figure 2). We found that the expression of both MCM proteins in tumor tissues was significantly upregulated. Moreover, the correlation between MCM level and HCC stages was also analyzed in GEPIA, which indicated that both MCMs were closely associated with HCC stage (Supplementary Figure S1).

**Figure 3:**
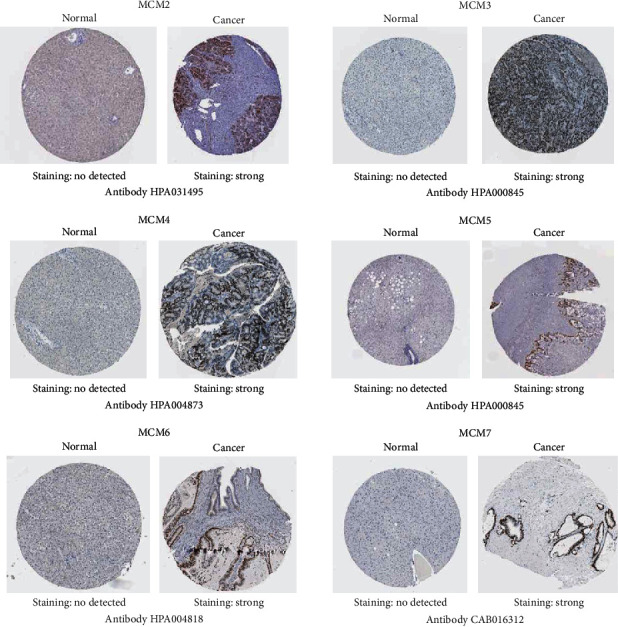
The protein expression of MCMs in HCC shown by immunohistochemistry staining images based on the Human Protein Atlas.

In addition, the immunohistochemistry (IHC) staining images for MCM protein in HCC and normal liver tissues were extracted from the HPA database (Figure 3). We found that these proteins were both significantly increased in HCC tissues compared to normal liver tissues.

### 3.2. Prognostic Values of MCMs in HCC Patients

Then, we utilized the database of KM plotter to analyze the correlation with high expression of MCM2 and worse RFS (HR = 1.73, *P* = 0.001). HCC patients with high level of MCM3 showed unfavorable RFS (HR = 1.81, *P* = 0.00063). High expression of MCM4 had poor RFS (HR = 1.51, *P* = 0.02). High levels of MCM5 were correlated with decreased RFS of HCC patients (HR = 1.89, *P* = 0.00018). Moreover, the level of MCM6 (HR = 2.08, *P* = 0.000012) and MCM7 (HR = 1.78, *P* = 0.0019) was also associated with RFS in HCC patients, respectively (Table 2).

The further analysis of these impacts on prognosis by these proteins is provided. We found that the prognosis significance of MCMs had a close correlation with some clinicopathological parameters, including clinical stages, pathology grade, and vascular invasion (Table 3). High expression of MCM2 was prominently associated with worse OS in HCC stage 1+2 (HR = 2.13, *P* = 0.0019) and stage 3+4 (HR = 2.09, *P* = 0.043). Likewise, similar results on stage 1+2 and stage 3+4 were also observed in MCM3 (HR = 1.85, *P* = 0.0152; HR = 1.77, *P* = 0.0672), MCM4 (HR = 1.61, *P* = 0.0669; HR = 3.06, *P* = 0.000093), MCM5 (HR = 1.92, *P* = 0.009; HR = 1.84, *P* = 0.0357), MCM6 (HR = 2.34, *P* = 0.0007; HR = 2.24, *P* = 0.0081), and MCM7 (HR = 2.13, *P* = 0.0019; HR = 2.09, *P* = 0.043).

Both OS significance of MCMs had a significant correlation with no vascular invasion, which indicated that high level of MCMs could predict the poor prognosis in HCC patients without vascular invasion. In the HCC patients with vascular invasion, only MCM7 could suggest a poor prognosis (HR = 2.27, *P* = 0.0328). Furthermore, the OS significance of MCM2 was not associated with grade 1 (HR = 2.43, *P* = 0.0666), but was associated with grade 2 (HR = 1.82, *P* = 0.0368) and grade 3 (HR = 4.13, *P* = 0.000088). Other MCMs are well summarized in Table 3. Next, we analyzed the association between prognosis significance of MCMs and hepatitis virus infection and alcohol consumption, respectively. The results indicated that both MCM overexpression had a positive association with poor OS in HCC patients without hepatitis virus infection or alcohol consumption. Only MCM5 and MCM7 had a significant correlation with poor OS in HCC patients with alcohol consumption. MCM6 could predict the poor OS (HR = 2.04, *P* = 0.0316), RFS (HR = 1.74, *P* = 0.0258), PFS (HR = 1.7, *P* = 0.0218), and DSS (HR = 2.26, *P* = 0.0469) in HCC patients with hepatitis virus infection. The detailed results are summarized in Table 4.

### 3.3. MCM Genetic Alteration in HCC Patients

**Figure 4:**
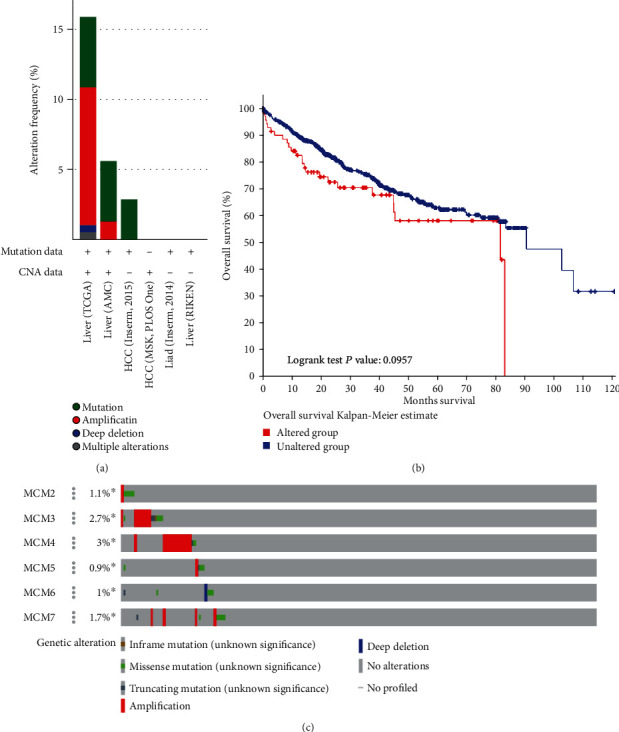
MCM alteration in HCC (cBioPortal). (a) MCM genetic alteration in TCGA firehose legacy datasets, AMC hepatology 2014 datasets, and INSERM Nat Genet 2015 datasets. (b) Kaplan-Meier plots comparing OS in HCC patients with or without MCM genetic alterations. (c) Alteration frequency of MCMs based on the cBioPortal dataset.

By using the cBioPortal database, we found that the percentages of MCM genetic alterations were 15.92%, 5.63%, and 2.88% in three datasets, including INSERM, AMC, and TCGA (Figure 4(a)). However, we analyzed the correlation between MCM gene alterations and survival outcome, which indicated that cases of MCM gene modification were not associated with OS (*P* = 0.0957, Figure 4(b)). The alteration frequency of MCM2, MCM3, MCM4, MCM5, MCM6, and MCM7 was 1.1%, 2.7%, 3%, 0.9%, 1%, and 1.7% based on six datasets, including MSK, INSERM, MSK, AMC, RIKEN, and TCGA (Figure 4(c)).

### 3.4. Correlation Analyses of MCMs in HCC Patients

**Figure 5:**
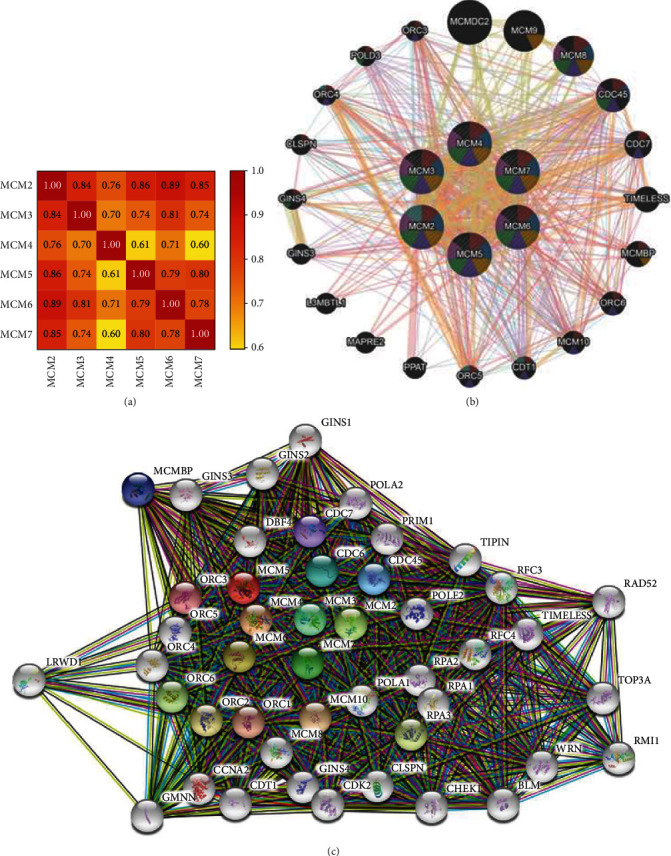
Coexpression and interaction of MCMs at the gene and protein levels in HCC patients. (a) Spearman's correlation analysis of MCMs. (b) The interaction network among MCMs at the gene level based on the GeneMANIA dataset. (c) The interaction network among MCMs at the protein level based on the STRING dataset.

We extracted the MCM mRNA level between each other in HCC from TCGA Provisional dataset (RNA Seq V2 RSEM) by using the cBioPortal. Spearman's correlation analysis among these MCM levels suggested significantly positive correlation between both MCM2/3/4/5/6/7 and other MCMs (Figure 5(a)).

Next, we utilized the GeneMANIA tools to analyze the association of MCMs at gene level (Figure 5(b)). This result indicated that the physical interactions among MCM2-7 were significant in this network, which might attribute to the shared protein domains. Relationships were significantly found among MCM2-7 in coexpression. Furthermore, pathway was noticed in reactome among MCM2-7 and other key genes, including CDC45, CDC7, ORC6, MCM10, CDT1, ORC5, GINS4, CLSPN, ORC4, and POLD3.

**Figure 6:**
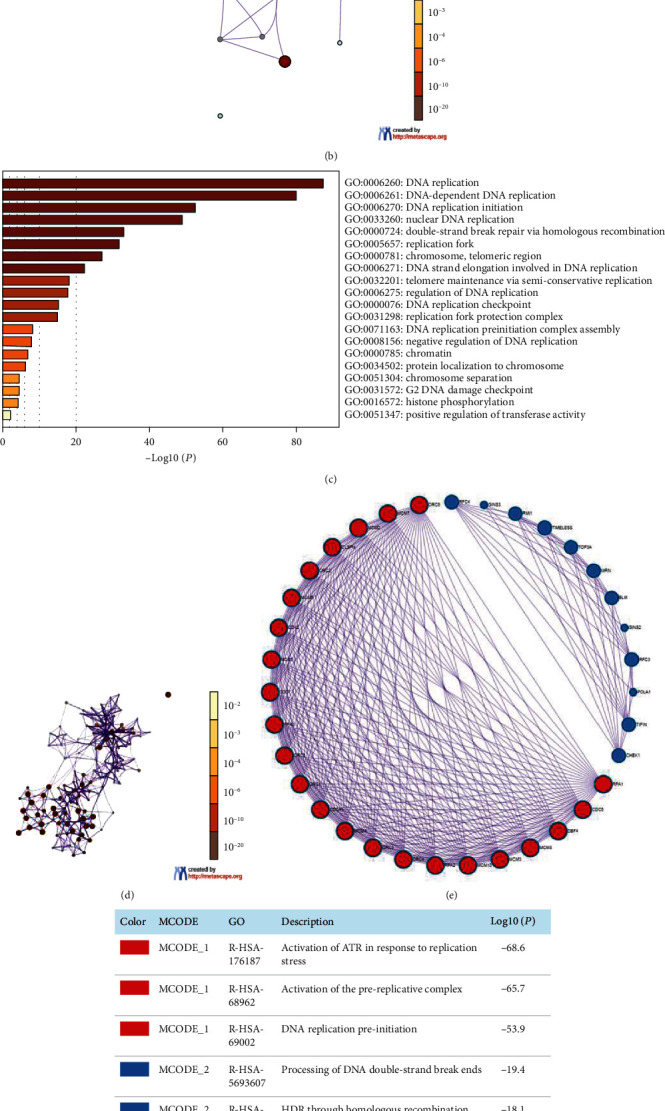
Gene Ontology (GO) and Kyoto Encyclopedia of Genes and Genomes (KEGG) enrichment analysis of MCMs and neighboring genes in HCC patients. (a) Top 5 KEGG enrichment. (b) Network of KEGG enriched terms. (c) Top 20 GO enrichment. (d) Network of GO enriched terms. (e) Protein-protein interaction (PPI) network by the Metascape database. (f) PPI network by functional enrichment analysis based on MCODE components.

We further identified the protein interactions of MCM2-7 by the STRING database (Figure 5(c)). The interactions among MCM2-7 were shown in experiments, databases, and coexpression. Moreover, the network for MCM2-7 and the 40 altered neighboring genes was constructed, such as MCMBP, GINS3, GINS2, GINS1, POLA2, CDC7, DBF4, PRIM1, CDC6, ORC3, LRWD1, ORC4, ORC5, CDC45, TIPIN, POLE2, RFC3, ORC6, ORC2, ORC1, GMNN, CCNA2, CDT1, MCM8, MCM10, POLA1, RPA2, RFC4, TIMELESS, RAD52, RPA1, RPA3, GINS4, CDK2, CLSPN, CHEK1, BLM, WRN, RMI1, and TOP3A. The detailed results are shown in Figure 6(c).

### 3.5. Functional Enrichment Analysis of MCMs in HCC

Finally, we excavated GO and KEGG pathway data for MCMs and their 40 altered neighboring genes by using Metascape. Top 5 KEGG pathways were DNA replication, cell cycle, homologous recombination, pyrimidine metabolism, and viral carcinogenesis (Figures 6(a) and 6(b)). Top 20 GO enrichment are shown in Figures 6(c) and 6(d). Biological processes are as follows: DNA replication, DNA-dependent DNA replication, DNA replication initiation, nuclear DNA replication, double-strand break repair via homologous recombination, DNA strand elongation involved in DNA replication, telomere maintenance via semiconservative replication, regulation of DNA replication, DNA replication checkpoint, DNA replication preinitiation complex assembly, negative regulation of DNA replication, protein localization to chromosome, chromosome separation, G2 DNA damage checkpoint, and histone phosphorylation; cellular components are as follows: replication fork, chromosome, telomeric region, replication fork protection complex, chromatin, and centrosome. Next, the protein-protein interaction enrichment analysis revealed that biological functions were mostly connected with the activation of ATR in response to replication stress, activation of the prereplicative complex, DNA replication preinitiation, processing of DNA double-strand break ends, HDR through homologous recombination (HRR) or single-strand annealing (SSA), and homology directed repair (Figures 6(e) and 6(f)).

### 3.6. Expressions of MCM2-7 Were Increased in HCC

**Figure 7:**
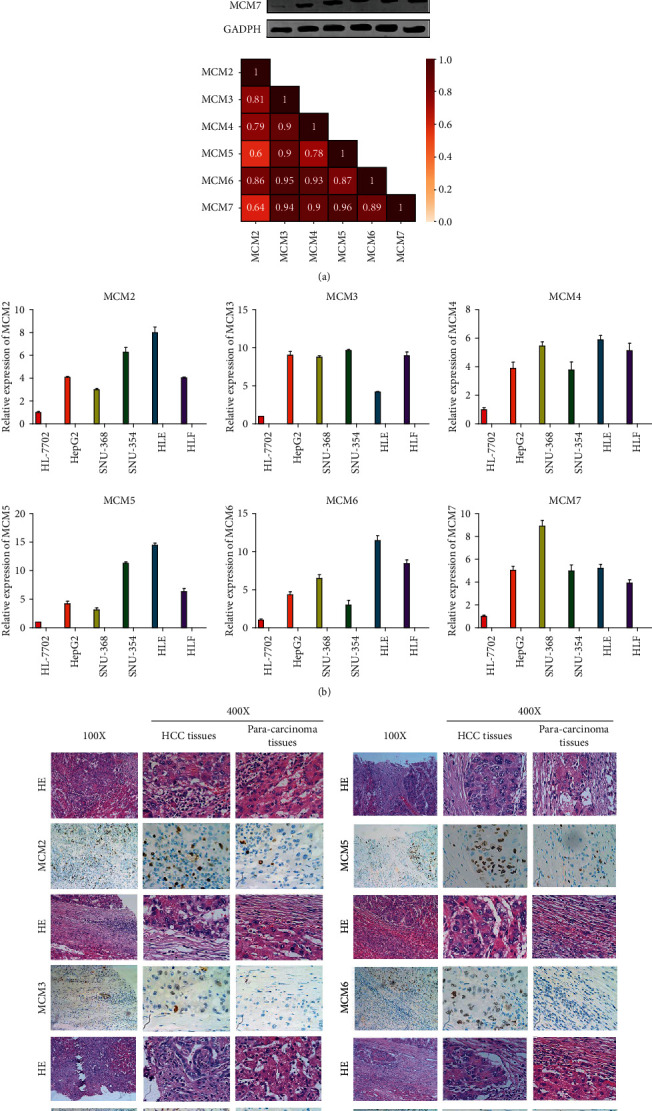
Identification of MCMs expressed in HCC cell lines. (a) The expression of MCMs in HCC cell lines and normal hepatocyte cell line and the correlation among MCMs in HCC cell lines. (b) The mRNA levels of MCMs in HCC cell lines and normal hepatocyte cell line. (c) The expression of MCMs in HCC tissues. High-magnification microscopic appearances of HCC tissues and paracarcinoma tissue are both taken from the low-magnification microscopic appearance by microscopes.

To further demonstrate the level of MCM2-7 in HCC, we detected the levels of MCM2-7 in several HCC cell lines and normal hepatocyte cell lines. The results indicated that the levels of MCM2-7 were increased in HCC cell lines (HepG2, SNU-368, SNU-354, HLE, and HLF) compared to the normal hepatocyte cell line (HL-7702). As shown in Figures 7(a) and 7(b), the expression of MCM2-7 in HCC cell lines significantly enhanced in the mRNA and protein level, which was consistent with the database analysis. Furthermore, the correlation analysis among these MCMs also suggested the significant positive correlation between these MCMs in HCC cell lines and normal hepatocyte cell line in the protein level (Figure 7(a) and Supplementary Figure S2). The next section of the survey was concerned with MCM expression in HCC tissues. The results, as shown in Figure 7(c), indicated that MCMs were significantly enhanced in HCC tissues compared to paracarcinoma tissues. Together, these results provided important insights into the fact that MCMs might play significant roles in the formation, development, and progression of HCC.

## 4. Discussion

Currently, more and more studies indicated that ectopic expression of MCMs could promote DNA replication [12] and accelerate cell cycle [11] and metastasis [14]. MCMs were involved in the development and progression of many human diseases [59]. In previous studies, the heterohexameric complex composed of MCM2-7 has been well summarized in human cancer cells. However, there are still many questions to be systematically solved about the expression, function, interaction, and prognostic value of MCMs in HCC. Therefore, we conducted a comprehensive analysis to reveal the transcriptional level, function enrichment, gene/protein interaction, and prognostic values of MCM2-7 in HCC.

In this study, we found that the expression of MCM2-7 was significantly increased in HCC tissues compared to normal liver tissues. MCM2, an oncogene, was correlated with the development and progression from cirrhosis to HCC [60]. MCM2 protein strongly expressed in high-grade squamous intraepithelial lesion may be useful as a cascade screening tool for detecting precancerous changes in cervical cancer [61]. Our results indicated that high level of MCM2 was significantly associated with worse OS/RFS/PFS/DSS in HCC patients without hepatitis virus infection. Zhao et al. revealed that MCM3 was a better marker of proliferation than Ki67, making it a valuable prognostic tool independent of ER and HER2 states [62]. In the group of nonalcohol consumption, our study indicated that HCC patients with elevated MCM3 expression had a bad OS/RFS/PFS/DSS. Choy et al. indicated that MCM4 could be used as a more sensitive proliferative marker for the identification of esophageal lesions [63]. We found that HCC patients with elevated MCM4 mRNA levels had unfavorable RFS and OS. Gong and his colleagues revealed that ectopic expression of MCM5 had a close correlation with malignancy and poor prognosis, which might be a potential prognostic marker in renal cell carcinoma [64]. In the study, we found that high level of MCM5 had also close correlation with the poor prognosis of HCC, especially in OS/RFS/PFS/DSS. Liu et al. suggested that MCM6 could indicate poor prognosis and promote migration and invasion, which could be predicted preclinical early recurrence in HCC patients to indicate more careful monitoring and aggressive treatment intervention [14]. Similarly, we also found MCM6 expression predicted poor OS and PFS in stage 1+2, stage 3+4, grade 1/2/3, or nonvascular invasion patients. Furthermore, MCM7 has advantages over traditional cell cycle markers, such as Ki67 and PCNA, because it has a higher sensitivity and is less susceptible to external factors, including inflammatory factors [65]. Given that Ki67 and PCNA expression can only be observed at certain stages of replication and can be easily interfered with, the presence of MCMs at all cellular stages may be the reason for the advantage [66]. Likewise, we analyzed the correlation between MCM7 level and prognosis, which indicated that MCM7could also be a valuable prognostic marker for HCC patients.

The relationship between MCMs and genetic alteration was observed in HCC using the cBioPortal database. However, the HCC prognosis was independent of the mutation, which might indicate that ectopic expression of MCMs was induced by other ways in HCC, such as protein phosphorylation, slunoylation, and ubiquitination. The epigenetic modifications, such as DNA methylation, histone acetylation, and noncoding RNA regulation, were still unclear in the MCM complex. HCC, as an acquired disease, may be more due to the epigenetic modifications and abnormal molecular signal transduction than to genetic factor and gene alteration [4, 5]. Therefore, further analyses of protein and epigenetic modification are needed for the comparison with the analyses of gene alteration. The mechanism of gene alteration of these MCMs also needed further exploration.

Furthermore, our results suggested that there was a significant positive correlation between MCM proteins which were both increased in HCC compared to normal liver tissues. GeneMANIA analysis revealed that MCM2-7 had a close association with CDC45, CDC7, ORC6, MCM10, CDT1, ORC5, GINS4, CLSPN, ORC4, and POLD3 at the gene level. These interactions were involved in reactome. STRING analysis also indicated that MCM2-7 had a significant correlation with MCMBP, GINS3, GINS2, GINS1, POLA2, CDC7, DBF4, PRIM1, CDC6, ORC3, LRWD1, ORC4, ORC5, CDC45, TIPIN, POLE2, RFC3, ORC6, ORC2, ORC1, GMNN, CCNA2, CDT1, MCM8, MCM10, POLA1, RPA2, RFC4, TIMELESS, RAD52, RPA1, RPA3, GINS4, CDK2, CLSPN, CHEK1, BLM, WRN, RMI1, and TOP3A, which forms an important network to perform a series of pathophysiological functions at the protein level. Wen et al. constructed a network in association with small cell lung cancer by bioinformatics analysis, indicating that the interactions among MCM2/3/6 and other hub protein were involved in carcinogenesis [67].

In order to further explore the related functions and signaling pathways of these proteins, we studied the functional enrichment of MCMs and its mechanism by Metascape. The results indicated that the pathways involved in MCMs might contain DNA replication, cell cycle, homologous recombination, pyrimidine metabolism, and viral carcinogenesis. These pathways were frequent disorder in carcinogenesis. For example, Lin and his colleagues found that DNA replication could accelerate the cell cycle to promote carcinogenesis by the MCM complex [68]. Breast cancer type 1 susceptibility protein (BRCA1), a tumor suppressor, induces DNA double-strand break repair by homologous recombination, protecting DNA replication forks from attrition [69]. Sweeney et al. found that the combination of glutamine and glutamine-derived metabolites in purine and pyrimidine synthesis was inhibited by dimethylaminopurine and rhodoxin, effectively blocking the key biosynthetic pathway for the survival of leukemia cells [70]. Viral carcinogenesis has been demonstrated in nasopharyngeal carcinoma [71] and HCC [72] by EBV and hepatitis virus infection, respectively. However, more work and experiments are needed to verify these bioinformatics predictions, which will help to investigate the role of MCM2-7 and related signaling pathways in the development of HCC.

## 5. Conclusion

In this study, we systematically summarized the mRNA and protein level of MCMs and useful prognostic information about MCMs in HCC. Furthermore, we also analyzed the genetic alteration, coexpression, gene/protein network, and GO/KEGG enrichment analysis of MCMs. Relevant results indicated that the mRNA and protein level of MCMs was significantly increased in HCC tissue compared to normal liver tissues. KM plotter analysis showed that high expression of MCMs indicated a worse OS/RFS/PFS/DSS in HCC patients. Importantly, HCC patients with MCM alteration did not display worse OS compared with the ones without MCM alteration, which might be attributed to ectopic expression of MCMs mediated by other molecular mechanisms. In conclusion, MCMs could be an effective prognostic marker for HCC. Our results can help to better understand the pathogenesis of HCC and develop more effective clinical treatments in the future.

## Figures and Tables

**Table 1:** Differential expression analyses of MCMs in HCC.

Gene	Database	Normal (cases)	Cancer (cases)	Fold change	*t*-test	*P* value	Reference
MCM2	Roessler Liver	Normal liver (21)	Hepatocellular carcinoma [22]	3.252	7.289	3*E*-08	[57]
	Roessler Liver 2	Normal liver (220)	Hepatocellular carcinoma (225)	3.144	21.853	2.7*E*-64	[57]
MCM3	Roessler Liver	Normal liver (21)	Hepatocellular carcinoma [22]	2.953	7.55	1.5*E*-08	[57]
	Roessler Liver 2	Normal liver (220)	Hepatocellular carcinoma (225)	3.023	23.777	5.7*E*-72	[57]
MCM4	Wurmbach Liver	Normal liver [10]	Hepatocellular carcinoma [35]	2.67	5.936	2.5*E*-07	[3]
	Roessler Liver	Normal liver [21]	Hepatocellular carcinoma [22]	2.541	6.958	6*E*-08	[57]
	Roessler Liver 2	Normal liver (220)	Hepatocellular carcinoma (225)	3.044	22.497	5.3*E*-66	[57]
MCM5	Roessler Liver	Normal liver [21]	Hepatocellular carcinoma [22]	3.353	8.167	3.9*E*-10	[57]
	Roessler Liver 2	Normal liver (220)	Hepatocellular carcinoma (225)	2.752	20.76	8.8*E*-64	[57]
MCM6	Roessler Liver	Normal liver [21]	Hepatocellular carcinoma [22]	3.353	8.167	3.9*E*-10	[57]
	Roessler Liver 2	Normal liver (220)	Hepatocellular carcinoma (225)	2.752	20.76	8.8*E*-64	[57]
	Chen Liver	Normal liver (76)	Hepatocellular carcinoma (104)	2.023	7.755	4*E*-13	[56]
MCM7	Roessler Liver	Normal liver [21]	Hepatocellular carcinoma [22]	2.453	7.019	2.7*E*-08	[57]
	Roessler Liver 2	Normal liver (220)	Hepatocellular carcinoma (225)	2.154	19.734	1.6*E*-57	[57]

*P* values ≤ 0.05 were considered statistically significant.

**Table 2:** KM plotter showing the correlation between different MCMs and survival outcomes in hepatic carcinoma.

Gene	RNAseq ID	Survival outcome	No. of cases	HR	95% CI	*P* value	Low-expression cohort (months)	High-expression cohort (months)
MCM2	4171	OS	364	1.96	1.38-2.7	0.0001	71	38.3
		RFS	313	1.73	1.24-2.4	0.001	36.1	13.27
		PFS	366	1.87	1.34-2.6	0.0002	40.97	15.83
		DSS	357	2.34	1.49-3.6	0.00015	84.4	61.73
MCM3	4172	OS	364	1.8	1.25-2.5	0.0013	71	46.6
		RFS	313	1.81	1.28-2.5	0.00063	40.97	15.97
		PFS	366	1.86	1.36-2.5	0.000077	36.27	13.33
		DSS	357	2.47	1.5-4.06	0.00025	104.17	81.87
MCM4	4173	OS	364	1.9	1.31-2.7	0.00058	70.5	25.6
		RFS	313	1.51	1.06-2.1	0.02	34.4	13.27
		PFS	366	1.53	1.12-2.0	0.0068	29.3	12.87
		DSS	357	2.27	1.42-3.6	0.00043	84.4	49.67
MCM5	4174	OS	364	1.94	1.36-2.7	0.00019	70.5	30
		RFS	313	1.89	1.35-2.6	0.00018	37.23	12.87
		PFS	366	1.79	1.32-2.4	0.00014	29.77	11.47
		DSS	357	2.24	1.43-3.5	0.00031	84.4	56.17
MCM6	4175	OS	364	2.29	1.61-3.2	0.0000023	70.5	24.1
		RFS	313	2.08	1.49-2.9	0.000012	42.630	13.33
		PFS	366	2.11	1.56-2.8	0.00000059	36.27	11.97
		DSS	357	2.73	1.74-4.2	0.0000056	84.4	49.67
MCM7	4176	OS	364	1.93	1.36-2.7	0.00019	71	38.3
		RFS	313	1.78	1.23-2.5	0.0019	42.87	18.3
		PFS	366	1.86	1.33-2.6	0.00022	36.27	15.17
		DSS	357	2.64	1.52-4.5	0.00035	59.7	24.13

*P* values ≤ 0.05 were considered statistically significant.

**Table 3:** The correlation between MCMs and survival outcomes in pathology parameters of hepatic carcinoma.

Gene	Survival outcome	Stage 1+2		Stage 3+4		Grade 1		Grade 2		Grade 3		Grade 4		Vascular invasion -		Vascular invasion +	
		HR	*P* value	HR	*P* value	HR	*P* value	HR	*P* value	HR	*P* value	HR	*P* value	HR	*P* value	HR	*P* value
MCM2	OS	2.13	0.0019	2.09	0.043	2.43	0.0666	1.82	0.0368	4.13	9*E*-05			1.99	0.0113	2.21	0.0789
	RFS	1.66	0.0281	2.06	0.0311	1.68	0.288	2.65	6*E*-05	1.58	1.58 0.1737			1.72	0.0261	1.72	0.0922
	PFS	1.75	0.0082	2.06	0.02	0.02	0.0534	2.38	6*E*-05	1.39	0.2373			1.91	0.0101	1.43	0.2257
	DSS	5.96	0.0002	2.31	0.058	2.05	0.2312	3.9	0.0012	4.62	0.0007			2.79	0.0088	1.43	0.5532
MCM3	OS	1.85	0.0152	1.77	0.0672	3.14	0.0164	1.73	0.0373	1.6	0.1314			1.94	0.0147	0.77	0.5264
	RFS	1.66	0.0174	1.38	0.3721	3.6	0.0164	2.01	0.0049	2.06	0.0435			1.85	0.0138	1.74	0.0919
	PFS	1.75	0.0046	1.39	0.2206	2.91	0.0096	2.12	0.0006	1.79	0.0759			1.97	0.005	1.77	0.0547
	DSS	2.7	0.0079	1.78	0.1291	4.81	0.0084	2.28	0.016	2.02	0.0728			2.34	0.0264	2.43	0.166
MCM4	OS	1.61	0.0669	3.06	3.06	2.18	0.1172	1.72	0.0662	2.68	0.0008			1.82	0.0287	1.6	0.244
	RFS	1.23	0.382	2.21	0.0115	0.67	0.4853	1.67	0.0596	1.81	0.0346			1.74	0.0586	0.57	0.098
	PFS	1.34	0.1646	1.91	0.0168	1.58	0.2514	1.83	0.0112	1.62	0.0549			1.58	0.0521	0.68	0.2275
	DSS	1.88	0.0987	3.26	0.0006	4.53	0.018	2.34	0.0164	4.13	0.0002			2.18	2.18	0.6	0.392
MCM5	OS	1.92	0.009	1.84	0.0357	3.34	0.0293	1.69	0.0422	2.03	0.0189			1.93	0.0124	0.63	0.2394
	RFS	1.65	0.0192	1.84	0.0486	1.49	0.4876	2.53	0.0001	1.59	0.0872			1.74	0.0243	2.47	0.0051
	PFS	1.64	0.016	2.02	0.0129	2.14	0.0539	2.27	0.0002	1.49	0.1165			1.63	0.032	1.9	0.0276
	DSS	2.26	0.0205	2.6	0.0094	7.89	0.0052	1.58	0.1959	2.83	0.0068			2.01	0.0554	0.42	0.1087
MCM6	OS	2.34	0.0007	2.24	0.0081	2.72	0.0325	2.8	0.0001	2.71	0.008			2.07	0.0091	1.75	0.1493
	RFS	1.79	0.0056	2.66	0.0044	1.81	0.2221	2.56	0.0001	2.42	0.0043			2.06	0.0028	0.66	0.2216
	PFS	1.94	0.0005	2.12	0.0098	2.56	0.0184	2.62	8*E*-06	2.41	0.0029			1.97	0.0023	1.45	0.2057
	DSS	2.96	0.0017	2.34	0.0129	2.81	0.0861	3.95	3*E*-05	3.15	0.003			3	0.0209	0.56	0.2984
MCM7	OS	2.13	0.0019	2.09	0.043	2.43	0.0666	1.82	0.0368	4.13	9*E*-05			1.91	0.012	2.27	0.0328
	RFS	1.66	0.0281	2.06	0.0311	1.68	0.288	2.65	6*E*-05	1.58	0.1737			1.57	0.0913	1.55	0.171
	PFS	1.75	0.0082	2.06	0.02	2.12	0.0534	2.38	6*E*-05	1.39	0.2373			1.4	0.1539	1.76	0.0548
	DSS	5.96	0.0002	2.31	0.0587	2.05	0.2312	3.9	0.0012	4.62	0.0007			2.01	0.0853	1.9	0.2445

*P* values ≤ 0.05 were considered statistically significant.

**Table 4:** The correlation between MCMs and survival outcomes in hepatic carcinoma based upon the alcohol consumption and hepatitis virus status.

Gene	Survival outcome	Alcohol consumption -		Alcohol consumption +		Hepatitis virus -		Hepatitis virus +	
		HR	*P* value	HR	*P* value	HR	*P* value	HR	*P* value
MCM2	OS	2.43	0.00022	2.09	0.0578	3.37	0.00001	1.73	0.1849
	RFS	2	0.0018	2.25	0.012	3.17	0.0000054	1.33	0.2661
	PFS	1.85	0.0024	2.37	0.0031	2.67	0.000022	1.45	0.1802
	DSS	3.4	0.000043	1.77	0.1096	3.25	0.000032	2.55	0.0326
MCM3	OS	1.87	0.0153	1.77	0.0696	2.69	0.000055	1.52	0.2548
	RFS	2.1	0.0012	2.18	0.0447	2.37	0.0007	1.85	0.0272
	PFS	2.28	0.0004	1.77	0.0412	2.53	0.000024	1.8	0.028
	DSS	3.66	0.0009	1.78	0.1115	4.07	0.000011	2.76	0.0547
MCM4	OS	2.34	0.0004	1.51	0.203	3.09	0.0000021	1.52	0.2329
	RFS	1.3	0.2924	2.25	0.014	3.32	0.0000053	0.64	0.1563
	PFS	1.43	0.1033	1.87	0.0168	3.12	0.00000047	0.75	0.2438
	DSS	3.22	0.0002	2.43	0.0126	5.01	0.000000011	2.9	0.0721
MCM5	OS	1.74	0.0208	2.59	0.0028	2.3	0.0004	1.87	0.0582
	RFS	1.9	0.0047	2.48	0.0024	2.94	0.000017	1.32	0.2703
	PFS	1.74	0.0078	2.22	0.002	2.57	0.000018	1.3	0.2851
	DSS	2.56	0.0019	2.78	0.005	2.79	0.0002	2.32	0.0392
MCM6	OS	2.7	0.000019	1.66	0.1114	2.86	0.0000058	2.04	0.0316
	RFS	2.37	0.0002	2.24	0.0074	3.37	0.0000019	1.74	0.0258
	PFS	2.44	0.00002	2.41	0.001	3.48	0.000000019	1.7	0.0218
	DSS	4.28	0.00000063	1.93	0.067	4.5	0.00000013	2.26	0.0469
MCM7	OS	2.13	0.0019	2.56	0.029	3.07	0.0627	1.75	0.0889
	RFS	1.92	0.0035	3.21	0.0059	3.02	0.000038	1.41	0.2594
	PFS	1.95	0.0035	3.06	0.0014	2.99	0.0000035	1.59	0.1061
	DSS	3.62	0.0003	2.44	0.0627	3.95	0.00001	1.93	0.2238

*P* values ≤ 0.05 were considered statistically significant.

## Data Availability

The data used to support the findings of this study are available from the corresponding author upon request.

## Supplementary Materials

Supplementary Materials Supplemental Figure S1: the expression of MCMs in different clinical stages. Supplemental Figure S2: the correlation between MCMs.
