# Supplementary figures and images for: Identification of the DNA Replication Regulator MCM Complex Expression and Prognostic Significance in Hepatic Carcinoma
Source: Biomed Res Int. 2020 Sep 9;2020:3574261. doi: 10.1155/2020/3574261 (PMC7499325; doi:10.1155/2020/3574261)

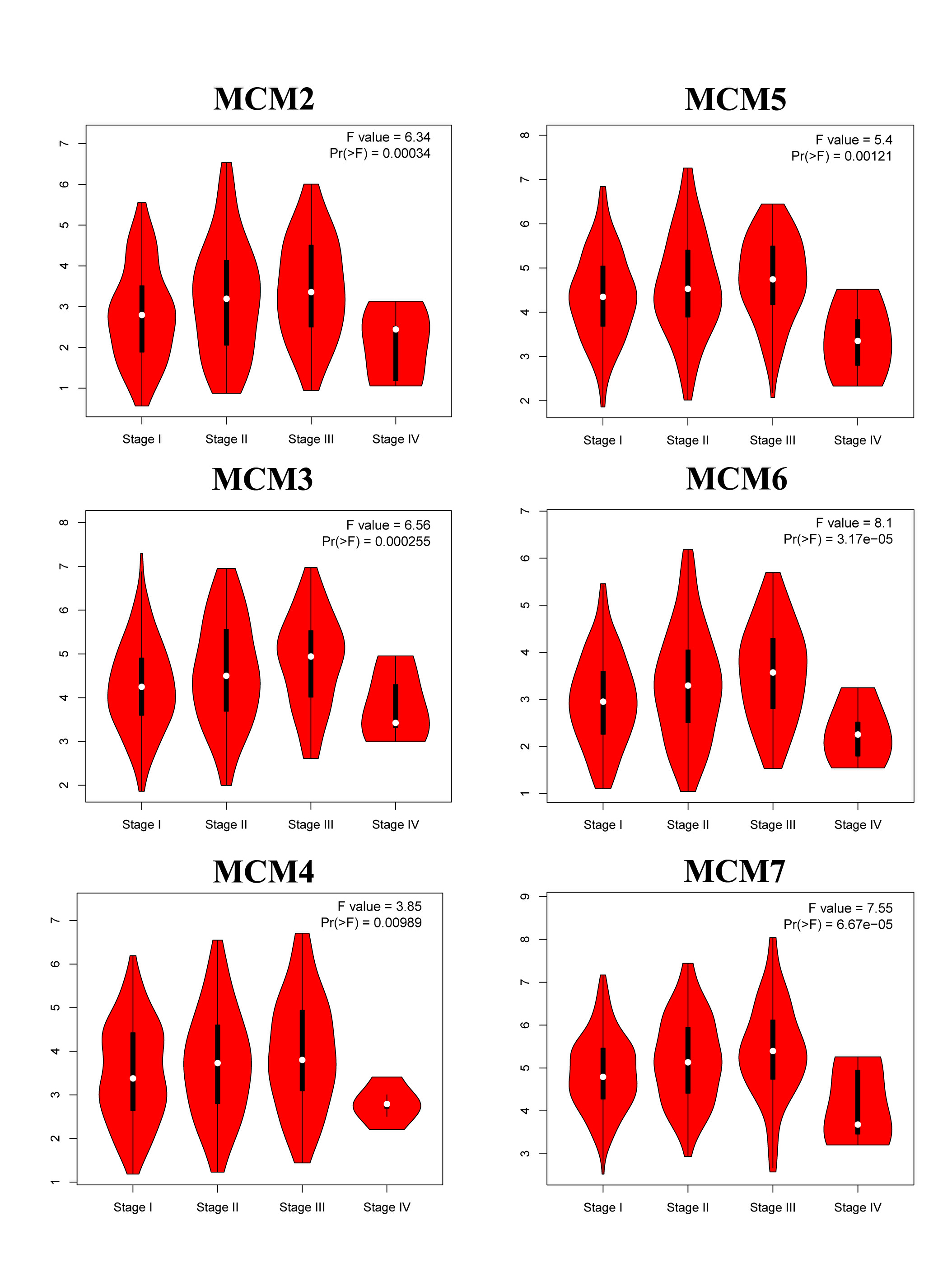

Supplement: Supplementary Materials — Supplemental Figure S1: the expression of MCMs in different clinical stages. Supplemental Figure S2: the correlation between MCMs. [file 3574261.f1.zip › Supplementary Figure. S1 (1).jpg]
